# From structure to substance: public participation as a scaffolding technology in citizen assemblies

**DOI:** 10.1057/s41599-026-06965-y

**Published:** 2026-03-19

**Authors:** Sofie Illemann Jæger, Julian;Iñaki Goñi

**Affiliations:** 1 Heriot-Watt University, Edinburgh, UK; 2 University of Edinburgh, Edinburgh, UK; 3 DILAB Engineering Design, School of Engineering & School of Design, Pontificia Universidad Católica de Chile, Santiago, Chile

**Keywords:** Science, technology and society, Politics and international relations

## Abstract

In this article, we assert that public participation events are best understood as scaffoldings that provide temporal infrastructures to citizens and that encourage “preferred ways” of acting within the participatory space. To exemplify this approach, we explore the citizens’ climate assembly (2022–2023) in Aarhus, Denmark as a case study of highly professionalised participation. Through a multi-method qualitative approach incorporating participant observation, focus groups, interviews, and surveys, we examine how both organisers and citizen members of the assembly perceive its design and implementation. In our results, we highlight two dimensions of the assembly’s scaffolding. Firstly, we analyse the guidelines that assembly organisers provided to signal what good interaction is within the assembly, namely, the Observation, Assessment, and Recommendation (OVA) method. This method shaped the affordances of members but also defined the epistemic hierarchies of contributions. Secondly, we analyse how the purpose of involving citizens in decision-making was constructed by assembly organisers and how it was communicated during the assembly, leading to uncertainties in process design and facilitation. Finally, we draw methodological and theoretical lessons from this case study and conclude that by treating participatory events as both structured and structuring, we can move beyond evaluations of participation as an ideal to be realised and instead investigate its situated practices and contestations.

## Introduction

It should be rather clear that the sort of communication that takes place within Public Engagement with Science and Technology (PEST) is not “natural” or how people would spontaneously behave outside the setting. Indeed, when we acknowledge that the communication occurring within PEST processes is *designed* (Goñi, 2024), we begin to notice how practices from asking specific questions to the roles, wording and procedures are crafted to bring about preferred forms of communication (Aakhus, 2007; Aakhus and Bzdak, 2015). Preferred likely by the organising body.

Over recent decades, institutions have faced mounting pressure to involve citizens in the governance of emerging technologies with the potential to reshape fundamental aspects of social life (Jasanoff, 2016). These technologies are diverse. For example, biotechnologies such as genome editing and cognitive enhancement raise profound questions about personal merit and individual autonomy (Hurlbut, 2017). Among them, climate change has sparkled particular interest (Willis et al., 2022).

PEST has been a prominent concern since the 1980s and 1990s (Sturgis and Allum, 2004). Within Science and Technology Studies (STS), this was reflected in the rise of the “public understanding of science” and “public communication of science and technology” movements. Participation is often framed as an “antidote” to earlier approaches that cast citizens as merely needing more education and scientific literacy to appreciate the social benefits of technoscience; the so-called “deficit model” of public understanding (Rowe et al., 2005).

Over time, institutional commitments to engagement have intensified, seeking to give more agency to citizens and extending from national initiatives to international frameworks, as exemplified by the OECD’s 2020 report *Innovative Citizen Participation and New Democratic Institutions: Catching the Deliberative Wave*.

It must be noted that ‘deliberative’ constitute a particular kind of participatory process (Bobbio, 2019). From the perspective of deliberative democracy, deliberation is best understood as the process reason-giving, critical questioning and mutual listening (Bächtiger and Dryzek, 2024). Specific formats, such as deliberative mini-publics try to foster mechanisms that support this kind of communication, by offering a ‘democratic mirror’ (Lafont, 2019) of the political community, as well an ‘epistemic filter’ (Lafont, 2019), that is, providing an informational infrastructure to help participants get to more considered conclusions (Bächtiger and Dryzek, 2024).

There are multiple ways to design deliberative mini-publics (Macnaghten, 2025; Curato et al., 2021). In the context of environmental policy, “climate assemblies” (Graham, 2024) are particularly salient. In climate assemblies, randomly selected, representative samples of the population are invited to participate in professionally facilitated sessions in order to deliberate about environmental issues, and typically, to arrive at policy recommendations (Lorenzoni et al., 2025).

Given the level of support and infrastructuring that goes into these processes, they have been compared to laboratory settings where “trained ‘experts’ design highly artificial spaces for deliberation, carefully protected and controlled from the wider world” (Macnaghten, 2024, 142). More broadly, STS scholars have recognised that all forms of structured participation inevitably face this challenge, given their inescapably designed nature (Goñi, 2024).

The fact that communication within PEST processes is designed also relates to the increasing professionalisation of public participation practitioners, especially in Western democracies (Barry and Legacy, 2023). In other words, there has been a rise of a new type of expertise in achieving desired forms of communication. In turn, the professionalisation and increasing technical language used to design participation have led to a standardisation of methodologies and formats, which also help expert practitioners position themselves in the market of participation (Voß and Amelung, 2016).

Nevertheless, then again, it is not the case that participation designs can mechanically force citizens to communicate in “preferred ways”. Citizens can accept, negotiate or reject designs (Soneryd and Sundqvist, 2022). It is also not the case that the ways in which people communicate during participatory processes will continue to endure once they leave the space. Academic research has noted increased knowledge and awareness of the issue and/or policy process as spillover effects of participation (van der Does and Jacquet, 2023). Still, the highly structured manner in which citizens communicate during PEST events is only appropriate in very few social spaces.

Finally, the design of participatory activities such as deliberative mini-publics operates across multiple levels of decision-making. Macro-level choices, such as the number of participants, thematic scope, communication mediums, and recruitment methods, typically attract the greatest attention (Curato et al., 2021). However, the micro-level decisions that shape the dynamics of interaction and collaboration, such as how rules of interaction are explained and enforced, receive far less analytical scrutiny, despite their significant influence on how participation unfolds in practice (Molinengo, 2023).

Against this backdrop, this article addresses the following research question: How do seemingly micro-level design choices of formal participation processes shape, enable, and constrain participants’ contributions to science and technology governance?

How, then, can we conceptualise the highly designed, yet always partial and temporal nature of public engagement? The central question of this study thus becomes: how can the concept of scaffolding provide an analytical lens for examining the ways in which participatory spaces encourage and structure “preferred ways” for citizens to participate?

Within engineering, scaffoldings are temporal infrastructures set up to sustain the construction, maintenance or repair of constructions. However, the concept of scaffolding has also gained popularity as a concept within learning theory. Within that space, scaffolding is understood as educational techniques that help learners perform a task with temporal outside support in the expectation that the temporal support will gradually be removed, thus encouraging autonomous performance (Wood et al., 1976; Upham et al., 2014).

To explore the potential of “scaffolding” as an analytical device, we draw on both STS theory around public engagement and sociocultural learning theory around scaffolding and mediation. Briefly put, we assert that public participation events as scaffolding are best understood as a temporal infrastructure that encourages “preferred ways” for acting and contributing in the participatory space.

## Theoretical framework

### Scaffolding within sociocultural learning theory

Within learning theory, the concept of scaffolding is typically associated with the theory of sociocultural development of Lev Vygotsky. Lev Vygotsky (1896—1934) was a Soviet-era psychologist and founder of cultural-historical psychology, who is considered one of the key authors of socio-constructivism. According to Wertsch (1985) there are three overarching themes in Vygotsky’s theory. These are the examination of the developmental origins of mental functions, the social and interactional explanation of higher mental processes, and the overall assertion that culturally constructed signs and tools mediate mental activity. In that sense, Vygotsky can be read as a systematic critique of the ontological and epistemological division between subject and society, between individual and collective development, and between biology and culture. Vygotsky’s concept of culture places special emphasis on the semiotic mediation of mental activity and individual participation in collaborative activities (Wertsch and Tulviste, 1992).

Central to Vygotsky’s theory is the notion of mediation. As Vygotsky asserted himself, ‘the central fact about our psychology is the fact of mediation’ (Vygotsky in Wertsch, 1985, 138). Mediation is the outcome of enculturation, of participating in culture. Through the use of cultural artefacts, human activity is re-shaped and regulated. It is mediated. For Vygotsky (1978), mediation does not imply that culture or history unidirectionally builds the mind, which would constitute a strong structuralist position, but that higher psychological processes are organised from the creative reconstruction of culture. For this reason, Daniels (2015) defines mediation as ‘the process through which the social and the individual mutually shape each other’ (34).

Based on Vygotsky’s idea of mediation, Wood et al. (1976) coined the concept of “scaffolding” to explain how humans learn to solve educational problems through external support. Wood et al. (1976) argued that tutoring involves more than just modelling and vicarious learning and that it ‘…involves a kind of “scaffolding” process that enables a child or novice to solve a problem, carry out a task or achieve a goal which would be beyond his unassisted efforts’ (90). Scaffolding is thus an external (cultural and social) structure of support that people learn by actively doing the task. This external support is meant to be gradually removed as the learner progressively internalises the task.

Interpretations of scaffolding have morphed over time across different educational studies and contexts but shared between different iterations remains the emphasis on scaffolding as a supportive device for producing a degree of learning in an individual that would likely not be achieved without it. Increasingly, learning studies have also focused on developing ideas of how scaffolding can be applied as a type of pedagogical ‘procedural facilitation’ that has the flexibility to be adapted to include different learning needs (Kheng and Siang, 2014, 181). This type of scaffolding, called ‘redundant scaffolding’, works through a parallel use of multiple scaffolds that are responsive to the need for different levels and types of support in a single situation (Kheng and Siang, 2014, 181).

### Extending the concept of scaffolding

While the idea of scaffolding originates from the work of Vygotsky and later learning theorists, we also take inspiration and critical perspectives from its recent applications in museum studies, where it has been adopted in participatory design (Simon, 2010; Kidd, 2016). In some ways, scaffolding can be likened to frameworks, but we use the idea of scaffolding to demonstrate its critical capacity as an analytical lens in participatory studies. The concept of scaffolding is an ordering structure that emphasises ‘the rules and resources that social actors draw on as they engage each other in social interaction’ and which ‘enable and constrain agency’ (Blaikie and Priest, 2017, 110-111). The notion of scaffolding also more clearly indicates a structure that is three-dimensional compared to frameworks that can be two-dimensional.

As such, we find that applying scaffolding helps draw our attention to the intersections of different components of the process. Crucially for this study’s focus, scaffolding also emphasises modularity, which is less clear in the idea of frameworks. A scaffold consists of different components, or modules. In the context of our study, such modules could include “remit-setting”, “intended outputs”, “stakeholder relationships”, and “participatory methods”. The way practitioners configure the relationships between these components, making up the “corners” in which they are joined together, determines how carefully they align and their ability to be responsive to changes in other modules.

This responsiveness will be discussed in greater detail shortly through the use of real-world examples. First, we want to illustrate how examining the modular capabilities of scaffolding allows us to deconstruct a scaffold to examine its different components and their significance. This includes considering the relationships between the different modules of the scaffold, and how they intersect with external domains of practice and knowledge, such as political systems, scientific expertise, or sociocultural contexts.

To clarify the shape and purpose of scaffolding participatory processes, we use an analogy from the work of Nina Simon, where a museum-goer is invited to take part in the painting of a mural:

If given the chance, very few people would opt to paint a mural on their own. The materials are not the barriers—the ideas and the confidence are. You have to have an idea of what you want to paint and how to do it (2010).

In an alternate version, the museum-goer is invited to contribute to the collaborative production of the mural but is given information about the overall purpose of the mural creation, why *they* were chosen, and how their contribution is meaningful. In other words, they know why they are participating and ‘what [they] are supposed to do to be successful’ (Simon, 2010, 23). Success is understood here as the participant being able to recognise the value of the overall project and how they can contribute to the collaborative process and its outcomes.

The scaffolding of the mural collaboration includes the material and conceptual lines given to participants, providing them with the resources necessary to contextualise the collaborative project in their own experiences. It helps show them that the project’s purpose aligns with their interests and how their ideas contribute meaningfully, even in areas where they may lack confidence, such as producing artwork or, as in this study’s context, understanding science policy.

As such, the notion of scaffolding helps illuminate how assembly organisers formulate objectives for their engagement activities and how the design of the participation format is aligned with the desired objectives. It also probes if and how practitioners communicate the engagement strategy to participants, including the objectives and the intentions behind the format. In line with Erving Goffman’s observation that an ‘individual’s framing of activity establishes its meaningfulness for [them]’ (Goffman, 1986, 343), it is important to ask how participants understand the objectives of the engagement and how they view their own role in achieving those objectives (Kidd, 2016, 61).

Moreover, investigating the scaffolding of participatory activities encourages scrutinisation of how organisations frame, facilitate, and police their participants’ interactions and discussions (Lynch and Alberti, 2010, 17). Asking how engagement strategies plan for the management of participant interactions is crucial for understanding how organisations might attempt to address existing power structures and social inequalities through their engagement programmes while simultaneously enacting their own agendas and ‘forms of power’ (Curato, 2019, 20).

Drawing from Activity Theory (Engeström, 2014; 2001), we understand participatory activities as complex, holistic, goal-directed, and historically shaped systems of human behaviour. Activities are dynamic processes of interaction between people and the environment. They consist of multiple organised actions, tools and signs, division of labour and rules, all of which work holistically towards a specific goal (Engeström, 2001).

From an STS perspective, the concept of scaffolding intersects with Susan Leigh Star’s (Star and Ruhleder, 1996; Star and Bowker, 2002) description of infrastructures. As Leigh Star (Star and Ruhleder, 1996; Star and Bowker, 2002) argued, no artefact is inherently an infrastructure; rather, infrastructures emerge when they operate in the background of social interactions, becoming embedded as support technologies for other practices. This invisibility, what she terms transparency, allows infrastructures to function as stable, often unnoticed components of social and technical systems.

Scaffoldings share this infrastructural element, and drawing from Leigh Star, we argue that scaffoldings cannot be reified ex-ante; instead, structural components of public participation become scaffolding when they are actively used to support desired forms of interaction. However, scaffolding differs in an important way from Leigh Star’s description of infrastructures: rather than sinking into the background, scaffoldings remain visible and are intentionally used in the open by assembly organisers to guide and structure participation. Much like in construction, these temporary frameworks do not aspire to invisibility but instead serve as explicit, provisional supports, shaping engagement while remaining conspicuously present in the participatory space.

In the rise of democratic and justice-oriented participation, there is, rightly, an emphasis on the re-distribution of power between participants and institutions. However, attempts to share power over co-creation cannot be equated with an absence of guiding lines that enable participants to understand their role in the process, providing them with opportunities for self-expression without the expectation or burden that they build the collaboration from scratch (Simon, 2010). While thoughtful guiding lines can thus help promote participant agency and autonomy—providing resources and contextualising the process’ relevance for participants—the trick becomes to balance these support beams against constraints on how participants are permitted to contribute.

In this study, we apply the concepts of scaffolding to highlight components of the process design that have the capacity to support, constrain, and, thus, shape the ways in which participants are able to contribute to tspecific participatory activities. Considering a recent citizens’ assembly where participants were invited to produce climate policy recommendations for their city, we will, for example, examine how participants understand the purpose of their contributions to the production of policy recommendations. We also consider one of the core methods that assembly organisers applied to structure citizens’ discussions, deliberations, and policy recommendations. In doing so, we investigate the intended and unintended effects that the ideas and practices this method prioritises have on participants’ experiences of their contributions.

## Methodology

The use of descriptive case studies is well-suited for investigations of ‘the complexity and uniqueness’ of social phenomena in a real-life context, such as the dynamics of interactions and contributions by the organisers and members of a citizens’ assembly (Simons, 2009, 21). It allows for the use of multiple methods through which to study a phenomenon from different perspectives (Thomas, 2020, 3–4). The focus we adopt on examining citizen participation as a process of collaborative knowledge-making practice calls for data collection methods that facilitate the capture of interaction and experience. The data collection consisted of participant observation, open-ended surveys, focus groups, and interviews.

Participant observations provided opportunities to investigate the dynamics of public participant groups, including interactions among participants and between participants and group facilitators. Guided by the outlined research question, we focus, in particular, on observations related to collaboration and deliberation. We used open-ended surveys with assembly participants immediately following their first meeting to identify important themes and directions for questions used in the subsequent focus groups and interviews.

Focus groups were subsequently conducted to examine relational questions and interactions between citizens (Morgan and Krueger, 1993, 7-11). Focus group participants were selected based on the citizens who had indicated in their survey response that they had an interest in participating in further data collection activities. The focus groups enabled us to expand on data collected from participant observations and themes that emerged from the open-ended surveys. The composition of each focus group was made to mirror the groups that citizens were members of in the citizens assembly. This composition was chosen as it meant that citizens already knew the other members of their focus group, and it was felt that the familiarity and trust they had already built as a group in the assembly would aid participants’ comfort with discussing sensitive or personal topics in the focus group.

Semi-structured interviews were conducted with both participants and facilitators in each of the case studies (Bryman, 2012, 468-470). Interviews explored more sensitive themes than were deemed possible or comfortable for participants during focus groups. For instance, we explored the perspectives of participant interviewees on institutional authority, their experiences of collaboration, and feelings of agency in their participation. Interviews with group facilitators and other organisers explored themes related to their construction and management of different components of the scaffolding. These included their internal strategies and objectives and external communication of aims, purposes, and parameters of participation to citizen participants (Hughes, 2017, 114-115; Connon, 2020, 103-104).

Guided by the study’s aim to examine the complex dynamics of assembly organisers’ and members’ ways of acting, we apply “Template Analysis” as our core analysis method. Template analysis is described as ‘a “style” of thematic analysis’ (Brooks et al., 2015, 218) to guide our data analysis. The epistemological position of template analysis, its proponents argue, is not bound to any one epistemology. The flexibility of the technique allows it to be adapted to the needs of a particular study and its philosophical foundations (Brooks et al. 2015, 205). The template analysis method places importance on tracing lateral and hierarchical relationships between themes (and between groups of themes), which makes the approach well-suited for exploring interaction and understanding knowledge as a relational practice. The method is generally intended to be applied to textual data, including, but not limited to, interview and focus group transcripts, open-ended questionnaires, or diary entries (Brooks et al. 2015, 203).

The full steps of template analysis can be briefly described as the creation of a preliminary coding template based on a priori coding, drawn from, for instance, concepts drawn from a literature review or the research questions, that is subsequently revised and expanded through its application to a subset of data (Brooks et al. 2015, 209). From the coding of the data subset, new codes are created based on themes emerging from the data and added to the template. Once a coding template has been created through the combination of a priori and bottom-up codes, the full template is applied to the remaining dataset. Examples of the coding schemes can be found in Appendix 1.

In recognition of its limitations, it is worth noting that while the case study approach offers a level of detail crucial for examining the complexity and nuance of human interactions and deliberative processes—essential for this study—depth in data collection is necessarily achieved at the expense of breadth. The specificities that give this case study its richness also present limitations: namely, they constrain the generalisability of the findings. This includes the influence of socio-cultural contexts and the particularities of the citizens’ assembly participatory format on research participants’ experiences and perspectives, as well as on the interpretive lens of the researchers.

### Case study

In the following sections, we examine how engagement practitioners, in conjunction with civil servants and policymakers, designed, communicated, and facilitated the scaffolding of a recent citizens’ climate assembly (2022–2023) in Aarhus, Denmark. Addressing the paper’s concern with how practitioners construct their scaffolding as infrastructure that supports and co-shapes participants’ behaviours and contributions, we explore how organisers of the Aarhus Climate Assembly (ACA) conveyed and managed the process’ purpose, participants’ envisioned role, and the intended outcomes of the engagement. Conceptualising practitioners’ strategies as scaffolding enables us to examine the intended structural logic of the participatory process design, and how this was later complicated by participants whose contributions filled, and sometimes reconfigured, the spaces between scaffolding components.

Understanding the scaffolding design, including its individual components, allows us to assess how participants’ behaviours and contributions align with or diverge from the spaces it sought to construct. Crucially, for this paper’s focus, the idea of scaffolding emphasises modularity. A scaffold consists of different components, or modules, that should be seen as interdependent parts of the process design, rather than as independently functioning parts. In the context of our study, such modules include “remit-setting”, “intended outputs”, “stakeholder relationships”, and “participatory methods”. The focus on these modules was chosen for their frequent appearance in data collection with participants, and for their interlinked roles within the scaffolding. The way practitioners configure the relationships between these components, making up the “corners” in which they are joined together, determines how carefully they align and their ability to be responsive to changes in other modules. We aim to illustrate that considering the modularity of scaffolding allows us to deconstruct it to examine the significance of its different components, their relationships, and how they intersect with external domains of practice and knowledge, such as political systems, scientific expertise, or sociocultural contexts.

Following a political commissioning of the Aarhus climate assembly in 2021, Aarhus Omstiller (“Aarhus Transitions”; AO), a subdivision of Aarhus Municipality’s local government, was tasked with coordinating the assembly process. They managed stakeholders, overseeing the relationship between policymakers, experts, citizens, and organisers from the Danish Board of Technology (DBT), who were to act as the designers and group facilitators of the process. Policymakers charged AO with carrying out the assembly. Their work included bringing the Board into the process, setting the overarching theme and questions of the assembly, and managing all communications with citizens and media. They also determined what parameters the process needed to follow to align with political requirements and oversaw the political handling of the assembly’s final recommendations.

The DBT was brought in as an independent third party to act as a ‘guarantor for the method and the independence of the process’ by designing the process and providing facilitation ‘at arm’s length’ to ensure the process would take place without political interference (ACA, 2023, 10-11). The team from the DBT first joined the project in February 2022, co-designing the process with members from AO while overseeing consultation with experts to inform the scaffolding of the assembly and identify suitable experts to engage with the citizens (observation notes (o.n.) 24.05.22; o.n. 21.06.22). During the implementation stages of the assembly, the DBT was tasked with training the group facilitators, who were members of AO, and leading the plenary sessions. These plenaries included “expert sessions”, feedback sessions between assembly sub-groups, brainstorming group exercises, and the final voting session. Moreover, assembly organisers from the DBT hosted several sessions throughout the process with guidance on how to ‘ensure a good dialogue’ in group deliberations (o.n. 08.10.22; o.n. 01.12.22).

Assembly members met five times between October 2023 and January 2024 to receive information about climate research and policy. Following the first meeting, practitioners allocated members into one of five sub-groups to deliberate on their policy recommendations. Additionally, some assembly members met with the City Council in November 2023 to provide an update to policymakers on the assembly’s process. Finally, in March 2024, assembly members met with City Council members at a public presentation event, where the official assembly report was handed over to the assembly’s two commissioning council members.

## Results

In order to explore the analytical usefulness of taking the perspective of scaffolding to unpack the technological underpinning of institutional public participation processes, we focus on two specific dimensions: the construction of interaction guides for citizens and the construction of explicit purposes of citizen involvement by assembly organisers.

On the one hand, we explore the structures that the organisers provided to citizens to guide how they are meant to deliberate on the topic. In this case, the OVA (“Observation, Vurdering, Anbefaling”; observation, assessment and recommendation) method. Through this lens, we are able to see how citizens internalised this particular way of talking, but also their resistance and re-framing of its value. In doing so, we were able to understand better how participation as a scaffolding afforded specific kinds of interactions and relationships between citizens and the ways in which citizens can position themselves across these affordances.

On the other hand, we explore how the purpose of involving citizens in decision-making was constructed by assembly organisers and how it was communicated. Moreover, we focused on how the construction of purpose informed, or did not inform, the design decisions behind the deliberative process. This allowed us to understand how participation as scaffolding shapes and is shaped by the construction of explicit objectives by organisers.

### **Scaffolds create affordances for specific kinds of relationships**

What is the difference between “observation”, “assessment”, and “recommendation”? (Citizen 20, o.n. 28.01.23).

“Observation” is neutral—the objective problem. “Assessment” is how you understand the situation and find it important. “Recommendation” is solution-oriented (ACA Practitioner 1, o.n. 28.01.23).

The above conversation between the main practitioner from the DBT and an assembly member during a plenary session referred to the core working method citizens were asked to structure their work around during the assembly, known as the OVA structure. Citizens were expected to move sequentially through these three steps for each sub-topic they chose to discuss under their allocated subject (for instance, “energy” or “transport”). Citizens were repeatedly asked to conduct their deliberations according to the appropriate step and its associated language and concepts. For example, they should first collectively produce “observations” about the climate question or issue at hand, reserving any comments relating to assessment, evaluation, opinion, or recommendation concerning the question for later discussions once the deliberations had progressed to this step of the OVA structure. It was often remarked by group facilitators, practitioners, and participants themselves that citizens needed to ensure that their written work should conform to language and ideas appropriate to the step they were addressing. For instance, for written contributions from the group regarding “observations”, citizens made an effort to speak of the issue through “objective” and “disinterested” statements that made reference, where relevant, to existing scholarly studies, data, and local policy.

This method was used during every deliberative session in the assembly’s sub-groups. What is surprising is that the citizen’s question was raised at the start of the *final* assembly meeting, following five meetings and four months of work. At this point, many other assembly members also admitted they were still confused about the structure, noting they had not been sure how to apply it during deliberations until they heard the explanation given during the final session (Group 2, 2023; Group, 3 2023; Group, 4 2023; o.n. 28.01.23).

What stands out about the practitioner’s response is the emphasis on identifying ‘objective’ problems and the weight placed on citizens to qualify their opinions of a problem through the performance of an ‘assessment’. Some citizens stated that they found the emphasis on a firm structure for conversations helpful, as they believed it could support momentum in a workflow intended to conclude with the production of specific outputs (Group 3, 2023; Group 4, 2023). One citizen observed how the structure encouraged ‘a quick focus on something relevant’ that would lend itself well to identifying tangible solutions (Group 3, 2023, 11). A member of the same group found the structure an ‘effective tool’ for producing a ‘product’ if used consistently.

However, they thought there should have been stricter control around the use of the structure: ‘[it should be that] when we say “observations”, we only mean “observations”, and then we *only* talk about [observations]’ (Group 3, 2023: 6). Similarly, one citizen observed that the structure provided by OVA could have prevented deliberations from going in circles. They indicated that their group’s lack of attention to the structure left them feeling ‘very frustrated’ because it resulted in a loss of momentum in arriving at solution-oriented policy recommendations (Group 4 2023, 23). The citizen stated that they felt compelled to step into the role of group facilitator at certain points, physically taking the OVA structure from the group facilitator to guide the discussion back to it and move their deliberations through the steps of the model (Citizen 23, 2023; Group 4, 2023: 23).

Citizens who felt mostly positive towards the structure spoke about its usefulness in the context of how it could support reaching a consensus about concrete recommendations and concern with efficiency in the face of limited time. A second noteworthy feature about the citizens favouring the structure was their familiarity with academic or data-driven approaches that move systematically from observation through to evaluation and conclusions based on “objective” knowledge. They suggested that they recognised the “workflow” process from their professional experience and found its recognisable structure useful (Group 2, 2023; Group 3, 2023; Group 4, 2023; o.n. 28.01.23). One such citizen thought that the structure had been unsuccessfully applied in practice, partly due to inconsistencies in its application during discussions, but that it could have been useful because ‘it fits the aim of communicating [our] recommendations to the city council and a political process’ (Citizen 28, 2023).

However, they believed that the structure’s ‘academic approach’ did not appeal to all assembly members because ‘not everyone has tried [that approach] previously and recognises its value’, resulting in a ‘“mish-mash”’ of deliberations because members did not understand or value the approach in the same way (Citizen 28, 2023). This citizen further found that despite their personal familiarity with academic approaches, they thought the structure created confusion about what was expected from citizens’ contributions and what was ‘one’s role in [the process] because you are confused about how you yourself are not an expert’ (Citizen 28, 2023).

They believed that the academic approach the structure encouraged citizens to adopt created an expectation that the citizens should produce academically grounded recommendations supported by expert research. Other assembly members echoed the citizen’s reflections. Most members from two other sub-groups expressed similar experiences of having “mish-mash” deliberations due to a lack of clarity about the structure and group members’ contradictory impressions of its value. During focus group discussions, members of these groups stated that the meaning and use of the structure had been ‘enormously muddied and unclear’ up until the final meeting when the DBT practitioner clarified its meaning ‘extremely clearly’ (Group 2, 2023: 10, 16). A member of the same group thought the structure in itself was problematic, not due to the lack of clarity around it, but due to how it made them feel restricted in their contributions:

I felt that the structure made perfect sense … [However], I did not understand what it was that they wanted me to do. I tried to adjust because I was expected to function within this particular structure, [even though] I disagree with it … I felt it was very difficult to find myself within [that structure] (Group 2, 2023: 16).

The same citizen thought the structure and its rhetoric created expectations for assembly members to be ‘highly educated’ rather than ‘very ordinary Danish citizens who have an opinion, a perspective on [climate policy]’. They believed it created an ‘unfair’ expectation for citizens’ contributions to conform to an academic, scientific way of working and wondered if it had been a factor in some group members choosing to leave the assembly because they found it ‘a frustrating process; *I* find it a frustrating process’ (Group 2, 2023: 16).

The group’s reflections on the significance of the academic complexity of the OVA structure are informed by their observations that assembly members who dropped out disproportionately represented citizens who left education after lower secondary education. For the final meetings, including the voting session, this resulted in a demographic overrepresentation of members who had completed upper secondary education and above (ACA 2023, 12–13; o.n. 28.01.23).

Their dissatisfaction was shared by a different sub-group who unanimously expressed frustration over the OVA structure, stating it was a ‘difficult way to work’ that caused them to use a ‘disproportionate amount of time’ working out what was classified as an observation, assessment, or recommendation (Group 5, 2023). They also felt that their recommendations might have been ‘sharper’, gaining more support during the final voting session, had they not been made less approachable by the level of detail and focus on rhetoric encouraged by the structure (Group 5, 2023).

Assembly organisers believed that the structure would serve as a tool to create a uniting foundation for a diverse set of people who are used to different working methods (ACA 2023, 51; ACA Practitioner 1 2023). Although some citizens supported the idea that OVA could help focus discussions on a common approach, their support was tempered by a feeling that they were inadequately equipped by assembly organisers and group facilitators to use it (Group 2, 2023; Group 3, 2023; Citizen 24, 2023). Moreover, some pointed to its rigid structure as being counter-productive at times, as it resulted in a premature narrowing of focus points for their recommendations (Citizen 16, 2023; Citizen 24, 2023).

The OVA structure meant citizens were, to an extent, limited to making observations about priority areas for climate actions at the very start of the process, as the structure prescribed a linear progression—from working on observations to assessments to recommendations—rather than encouraging an iterative movement between the three. The need for progressive rather than iterative workflows was closely tied to the limited time available for assembly meetings and the emphasis on producing tangible outputs. Thus, deliberations were purposely structured around OVA to make it a ‘process for the mind’ (ACA Practitioner 1, 2023).

The assembly organisers felt OVA would clarify at what points deliberations should address ‘factual’ issues and when they should discuss their ‘opinions’. They thought that by emphasising the OVA rhetoric and structure ‘often and insistently’, the division and clarity OVA brought to deliberations would make them more efficient and ‘self-organising’ (ACA Practitioner 1, 2023).

From a scaffolding perspective, one aspect that particularly stands out about the ideas informing the design and application of the OVA structure is that the linear aspects of the workflow conflict with the process’ intentions to highlight learning as a core tool and outcome of the assembly (AO, 2023, 1). Citizens’ learning, through iterative engagement with the perspectives of other members and experts, took place as a continuous process throughout the assembly. As such, their observations about climate policies were informed and developed throughout the assembly and not confined to its beginning. One citizen pointed to how OVA ‘restrict[ed]’ their group, preventing them from incorporating new ideas and opinions about which areas of climate policy to prioritise, because as soon as ‘you leave one level, it is as if you close the door to return to [observations about climate policy]’ (Citizen 24, 2023).

The inflexibility of the method required citizens to make early decisions about which climate issues to prioritise. This constraint impacted their ability to make informed choices, as they did not have the opportunity to iteratively discuss and share perspectives with other participants and stakeholders on what they believed were the most valuable or impactful areas of climate policy to influence. Scaffolds are not only determined by objectives, but they co-shape them.

Citizens assemblies are often highlighted for their potential to “empower” the voices of “ordinary citizens” by platforming their perspectives, making them known to policymakers and experts in a way that would likely otherwise not be possible (Harris, 2019). However, what those voices speak to greatly depends on what they are being asked. Here, we are not referring to an assembly’s topic or theme but rather to its objectives. While the immediate answer for many assemblies, including ACA, centres around the main aim to produce policy recommendations, much less is said, in ACA and elsewhere, about the purpose(s) of these recommendations (ACA, 2023; DBT, 2022).

Possible purposes of producing the recommendations which are suggested but rarely, if ever, clearly stated include:

- Are the recommendations meant to reflect new problems or questions related to climate action that were not previously identified in policy but have been highlighted by citizen contributions?
- Are they intended to incorporate new ideas for collectively addressing known problems—specifically, should they identify innovative solutions for climate action?
- Should the recommendations evaluate which solutions proposed by experts and stakeholders are most important to citizens?
- Will they reflect how citizens wish to prioritise climate actions against social needs, indicating the compromises they are willing or unwilling to make when social needs conflict with climate action?
- Finally, are the recommendations intended to provide a “sense-check” for existing policy proposals, thereby generating support that could help politicians pass climate action policies?

The many and varied possible intentions for citizen-produced recommendations carry implications for the entirety of the assembly scaffolding. The purpose informs how citizens understand organisers’ expectations for their contributions and how they understand their independent knowledge and experiences as being relevant to deliberations. The purpose underpinning the production of recommendations also sets demands for the design and management of relationships and interactions with other stakeholders, such as experts.

For ACA, individual assembly organisers, group facilitators, and citizens provided varied answers when asked what they understood the assembly’s purpose to be. One group facilitator stated:

I am not sure if I have ever asked that because I was not here [when the assembly was commissioned] … I think you should ask [the main organiser from AT] about it; he will definitely have a good answer to [that question]. I do not actually know (Facilitator 3, 2023).

The facilitator’s lack of knowledge about the purpose of the process aligns with responses from other group facilitators and assembly organisers. Although they expressed an understanding of the intended purpose, their answers were characterised by significant inconsistencies that, at times, made them contradictory:

I just think it’s been very unclear what the purpose was; I think that’s maybe where things went a bit wrong. It seems like that’s also what I hear when I talk to the citizens—that it was where things went wrong—and I think so too because I always felt that the purpose should be to give a voice to the people and come up with some solutions. That is the fundamental purpose of the whole process, but that has just been very unclear for us [the group facilitators], and actually, at certain points, we were a little uncertain about what we were really supposed to be doing. In the beginning, it was supposed to be …. that the whole process was about giving a clear voice to the citizens, where we were not supposed to interrupt them and so on. However, later on, it became a thing where we were actually allowed to interrupt [the discussion] if we had some climate facts or other angles [for them to consider] (Facilitator 4, 2023).

The group facilitators’ uncertainty about the purpose of the process affected how they understood their role in the process, including what kind of support they should provide to citizens. As is evident from the second facilitator’s statements, their changing perceptions, or increasing confusion, about the purpose were closely interlinked with how assembly organisers instructed group facilitators to moderate discussions.

As their understanding of what the citizens’ policy recommendations were supposed to reflect changed, they felt increasingly compelled to correct citizens’ contributions and offer information—‘climate facts’—for citizens to consider. The facilitator’s reflections indicate they went from believing the assembly should ‘[give] a clear voice’ to citizens, unmediated by group facilitators’ interference, to feeling that they had to occupy an advisory role to help bolster the factual foundation for citizens’ recommendations. The way their perception of the purpose influenced their approach to facilitation highlights the importance of having clear objectives, as well as the interdependent qualities of the different scaffolding components. Clarity about purpose affects the choices of assembly organisers and affects how citizens’ contributions are moderated.

The main assembly organiser from the DBT stated that their process design was not intended to favour either the production of innovative solutions or to function as a “sense-check” that would gather support for existing policy plans. They believe that it is rare that the level of complexity needed to achieve innovative solutions is realistic for a large collection of people, making citizens’ assemblies a less ideal format for recommendations striving to produce complexity (ACA Practitioner 1, 2023). Instead, the practitioner suggested that the assembly recommendations should fulfil two interconnected purposes: generate legitimacy for particular political solutions by clarifying which areas of climate policy citizens feel should be prioritised (ACA Practitioner 1, 2023). The first purpose expressed in this is the aim for citizens to *prioritise* climate policies; that is, to deliberate—and where necessary, negotiate—the order of priorities not only for competing climate policies, but also against the trade-off of prioritised for action for other areas of public policy. This could, for example, be where budgeting for climate policies conflicts with budgeting for policies targeting other social concerns and goals.

The second purpose expressed in the organiser’s statement—is to *establish* *legitimacy* for political decision-making, including existing proposals. This particular goal is also reflected in the toolkit guide produced by Aarhus Omstiller following the ACA. The guide outlines one of the process’ key purposes as ‘increasing the quality of political decision-making processes’ by creating ‘greater legitimacy for difficult political decisions through a demographic representativeness’ and ‘broad support from citizens with varied opinions and backgrounds’ (AO, 2023, 1).

The collective statements from assembly organisers and group facilitators about the purpose of ACA provide an opaque view of what motivated the assembly and what the aims were for citizens’ production of policy recommendations. Information about the assembly was shared publicly through the assembly website,^1^ the ACA policy recommendations report, and affiliated City Council websites. Across all these sources, several different aims for the recommendations were given. These include that ‘with the people’s support, it will become easier to pass climate policies with the least possible resistance’ (AC, 2022) and that ‘we must find the solutions [to the climate crisis] together … [and the assembly] will produce qualified suggestions concerning some of the big challenges we face’ (ACA 2023, 7).^2^

The Aarhus Council website also provides testimonies from the principal DBT practitioners, stating that the assembly is a process where citizens are introduced to themes and information from experts to inform their collective work as a group to ‘identify what they believe would be good solutions’ for climate challenges (AC, 2023).

Moreover, ethnographic observations from the assembly meetings showed a consistent pattern of participants working to identify what they found to be key issues for climate policy under their thematic area, work that was informed by expert presentations from the first meeting. Following their identification of issues during the “observation” stage, participants moved to assess the most significant aspects of the issue, discuss possible solutions—informed by expert presentations and independent research—and reflect on how such solutions intersected with their personal experiences as citizens. The final stage was reaching a consensus in the sub-group on a concrete recommendation for the Council.

When considering how assembly organisers determine the purpose and aims of their processes, it is important to note that multiple motivations and objectives may be sought simultaneously through an assembly. It is possible to pursue objectives to identify new climate solutions or generate support for existing climate policy proposals while also aiming to produce insights into how citizens wish to prioritise climate needs against social needs. We suggest, however, that there must be a strong sense of clarity about the process aims and a deliberate effort behind how the design of the scaffolding will support the achievement of these.

In ACA, the confusion that characterises many testimonies from practitioners, group facilitators, and citizens suggests that the process came before the purpose. When discussing the commissioning of the assembly, a principal DBT practitioner stated:

A funny thing about Aarhus is that when it was decided that we should carry out a citizens’ climate assembly in Aarhus, there wasn’t a topic; that is, it did not start with the topic, but with “citizen involvement”. That is pretty relevant, and I think that it is often the order in which it happens, and that also has to do with the fact that it is a relatively new method. It is rare that you have an issue and then you think, “We will use a citizens’ assembly”. It is, as a rule, more like “we have heard about citizens’ assemblies, so now we have to find an issue that we can use for it.”’ (ACA Practitioner 1, 2023).

In Aarhus, the attempt to fit the purpose to the process format was seen as less than ideal by the DBT organisers. Nonetheless, they perceived it as having been successfully accomplished. They believed they had managed to identify meaningful themes suitable for the assembly format, ‘[finding some issues where [they] felt like it ended up making sense’ (ACA Practitioner 1, 2023). However, there is a sense in this that purpose is the same as focus and that identifying relevant topics for the assembly would translate into having a sincere and purposeful motivation for commissioning it.

Moreover, neglecting to distinguish between “topic” and “purpose” results in inattention to how the nature of the purpose determines the demands placed on the process design. Such demands include how facilitation and models for collaboration between stakeholders govern how citizens are allowed and enabled to contribute. Significantly, it also comes to bear on how outputs from the assembly are received and treated by policymakers. The original motivation for the commissioning of the process helps determine policy considerations of its value for political decision-making:

For sure, the politics [around the motivation for the assembly] have been very unclear. I also think that is why [the processing of recommendations] is taking place so slowly now because they don’t really know what we [the Municipality] want; what we are going to use it for—because there hasn’t been any plan for it (Facilitator 4, 2023).

While the concept of citizen assemblies continues to gain ground in public engagement for climate policy and action, it should not be taken as an unambiguous concept that can be adopted in isolation from how it is informed and shaped by the local context of an assembly. This context crucially includes articulating the intended purpose(s) of the process. In Aarhus, the process scaffolding shifted between two focuses. At times, it emphasised integrating the values and knowledge of “everyday experts” into climate policy. At other times, it stressed that the process should prioritise observations and assessments grounded in factual knowledge over personal experiences.

Equally, public communication for the process, and at various times practitioners, spoke of identifying solutions, which was experienced as a burden for several citizens due to its associated expectations for expert knowledge, while practitioners internally indicated that they did not expect novel or complex knowledge outcomes or recommendations from the assembly.

The external portrayal of the process and practitioners’ suggestions at various points indicated that one value of citizens’ work was its potential alignment with existing policy plans. If their recommendations matched these plans, it would demonstrate public support for political efforts, enhance political legitimacy, and provide policymakers with a stronger mandate for climate policy.

Nonetheless, when asked about how such overlaps between recommendations and existing policy would be processed internally, lead organisers from AO stated that they believed that in such cases, the recommendation would simply be disregarded due to not having contributed something new to policymaking. It would not be registered and thus conceived of as a contribution and outcome in itself (Facilitator 1, 2023). The potential replication of work frustrated some citizens as they believed it diminished the significance of the time and effort they contributed to the process, making it “wasted” work (Group 2, 2023; o.n. 01.12.22).

Testimonies from AO regarding the political processing of the recommendations seem to support citizens’ fears that replication of work could be used as a political instrument to reassert power for existing political agendas rather than motivated by a sincere desire to ‘rethink authority’ and adopt a new model for integrating citizens’ opinions, experiences, and values into democratic decision-making for climate policy (o.n. 08.10.22). AO suggestions that recommendations aligning with existing plans could be entirely disregarded suggest that, at worst, citizen contributions could indeed become “wasted” in terms of policy outputs, not even receiving full political processing contrary to promises made by the commissioning authority (o.n. 08.10.22; Facilitator 1, 2023; ACA, 2023).

The confusion, hesitancy, and, at times, scepticism among group facilitators and citizens in ACA, which were produced by a lack of consistency in the process scaffolding, highlights the dangers of placing “process before purpose”. Discourse in academia, policy, and the engagement sector on the innovative and democratic potential of citizen assemblies must be accompanied by a critical assessment of when and why an assembly is a productive process for addressing particular policy aims.

On the ACA website, under the heading ‘Why will there be a citizens’ climate assembly’, assembly organisers attribute the motivation for commissioning ACA to a commitment from the Council in the city’s Climate Action Plan 2021–2024 ‘to test new methods for involving citizens in the green transition’ (AC, 2022). The democratic aims and potential of assemblies have been extensively discussed and remain a core consideration for commissioning authorities for how they can address “democratic issues” (Sandover et al., 2021; Elstub and Escobar, 2019). However, citizens’ experiences of confusion and disappointment in ACA evidence the risks that come with assemblies being adopted for democratic or political motivations without equal attention to how involving citizens in policymaking can meaningfully contribute to policy developments. Commissioning authorities risk assemblies being perceived as performative measures intended to reinforce political power and advance existing agendas if the engagement’s purpose is not clearly defined. Without a clear explanation of how citizens’ contributions will meaningfully inform climate policy aims, there can be the appearance of a lack of genuine concern for their voices and priorities.

## Discussion and conclusions

In this article, we discuss the implications of studying public participation events as scaffoldings that provide temporal infrastructure that encourages “preferred ways” for acting and contributing to the participatory space. This approach highlights both the artificiality or construction of interactions within the participatory fora, but also how it embeds specific kinds of normativity, both from the perspective of the assembly organisers of such events, and from the citizens as active users of this infrastructure.

In order to exemplify the analytical relevance of this approach, we studied the citizens’ climate assembly (2022–2023) in Aarhus, Denmark. This deliberative process was designed by the Danish Board of Technology (DBT) and is representative of highly professionalised deliberative contexts. For this reason, it offers a rich research site to more clearly unpack the structures that support these processes; that is, their scaffolds.

First, we turned our attention to the guidelines that assembly organisers provided to signal what good interaction is within the assembly, namely, the OVA method. The OVA structure functioned as a scaffold that shaped the affordances available to assembly members, supposedly helping citizens make distinctions among the categories of observation, assessment, and recommendation. In doing so, it implicitly delineated what counted as legitimate contributions, reinforcing a particular epistemic hierarchy within the assembly.

While some participants found the structured workflow useful for maintaining focus and producing tangible outputs, others experienced it as restrictive and ill-suited to the iterative nature of their learning and engagement. The assembly’s reliance on OVA highlights a tension between the intended facilitation of participation and the constraints it imposed on how citizens could articulate their perspectives. This case thus raises broader questions about how scaffolds of interactions configure participation—not merely by enabling deliberation but by legitimising specific modes of reasoning and expression over others.

Afterwards, we described how the purpose of involving citizens in decision-making was constructed by assembly organisers and how it was communicated during the assembly. While assemblies like ACA are framed as platforms for amplifying citizen voices, the nature of these voices is contingent on the assembly’s underlying purpose. However, ACA’s purpose was inconsistently articulated across organisers, group facilitators, and participants, leading to uncertainties in process design and facilitation. As demonstrated, the lack of clarity around the core purpose(s) of the assembly recommendations affected how citizens’ contributions were framed—that is, whether they were as expressions of lived experience, problem identification, or validation of existing policies. This, in turn, caused increased confusion and anxiety amongst citizens and group facilitators concerning how they were intended to navigate and apply the OVA interaction guide, as its structure was, at times, at odds with, for instance, expressions of lived experience or problem identification.

The effects of the ambiguity surrounding the purpose of the assembly process, including its interdependent relationship with other scaffolding components such as the interaction guide, underscore the performative nature of process design. In other words, scaffolds do not simply enable participation but shape what participation means in practice. The case of ACA highlights how the commissioning of assemblies often prioritises method over purpose, with implications for their legitimacy, the reception of citizen contributions, and the extent to which they can meaningfully inform policy.

These findings further enrich our understanding of participatory activities as complex, holistic, goal-oriented, and historically situated systems of human behaviour (Engeström, 2001; 2014). As outlined, the mediation of participation emerges through the dynamic interplay of specific rules, such as the OVA method, which structures divisions of labour between citizens and practitioners, and the participatory instruments employed to materialise this collaboration. Moreover, our results introduce nuance to the notion of goal-directedness in participatory activities. Rather than being solely driven by predefined remits and objectives, these goals appear to be co-produced through the methods themselves, suggesting a more flexible and loosely constructed orientation.

Future research might investigate deeper conceptual integrations of activity theory with the specific conceptualisation of scaffolding advanced in this article. For instance, further inquiry could examine how internal or external changes within participatory systems generate conflicts and dilemmas, such as those observed in the tensions arising from the enforcement of the OVA structure and the divergent descriptions of the activity’s remit. Activity theory posits that examining the manifestations of conflicts and dilemmas encountered by actors can provide a valuable lens through which to uncover the deeper historical contradictions that shape and situate activities (Engeström and Sannino, 2011). Investigating this dynamic offers promising avenues for reconnecting the micro-analysis of participatory events with their broader historical context. Moreover, it can illuminate the sources of resilience and the transformative potential of participatory practices without negating their messiness and complexity.

Participation is often discussed as an abstract and elusive category (Wynne, 2007), ranging from more idealised notions to concrete, institutionalised practices (Goñi, 2024). However, recent STS literature has increasingly recognised that participatory methods are highly orchestrated political spaces that rarely exist in the “wild” (Macnaghten, 2024). As Sarah Davies (2024) puts it, participatory mechanisms are themselves constitutive technologies of the issues they address and the publics they create. This perspective challenges the idea of participation as a neutral or transparent process and instead foregrounds its embeddedness within specific sociotechnical arrangements.

Rather than limiting our research, adopting the perspective of participation as a technology should open new avenues for investigation. It encourages a shift from evaluating participation in terms of its effectiveness or the explicit rationales behind it to understanding how it actively shapes its own normativity through use and not as only conceptual categories. Against this background, this study expands on this emerging STS perspective by demonstrating its empirical potential in studying practice.

On the one hand, our findings suggest that as a specific kind of technology, participation is best understood as scaffolding. That is, a temporary structure aimed at supporting desired ways of engaging. This metaphor helps to account for both the enabling and constraining aspects of design, but also how, in contrast to infrastructures that are made to sink into the background, these design elements are meant to be visible and are constantly reinforced by organisers. It also highlights its normative entrenchment. While scaffolding provides participants with structured pathways for engagement, it also defines the parameters within which their contributions are deemed valid or relevant.

Through this concept, we were able to observe how highly visible structures, such as the OVA method, create both affordances and sites of resistance. These scaffolds do not merely structure deliberation; they can become deliberation subjects in their own right as participants react to, negotiate, and sometimes challenge the ways in which their engagement is shaped. This perspective opens up new questions about how participatory scaffolding helps contest and are contested at the same time.

On the other hand, our findings reveal that adopting a scaffolding perspective invites a particular form of empirical inquiry. By focusing on the supporting structures provided by organisers, we were drawn to micro-sociological phenomena, such as the specific ways in which group facilitators translate methodological guidelines and purpose into practice, the moments when participants deviate from prescribed formats, or the subtle ways in which power is exercised through seemingly neutral procedural elements. Our experience suggests that an STS perspective on participation could benefit from closer attention to the minutiae and nitty-gritty of participation, identifying the technologies in use through detailed microanalysis. This approach enables a more granular understanding of how participatory events unfold in practice, highlighting the contingencies, frictions, and improvisations that shape their outcomes.

At the same time, it would be productive to explore how macro perspectives, such as recent research examining the institutionalisation of participation as a technology (Pallett and Chilvers, 2022), interact with this micro-level approach. If participatory infrastructures are themselves political and epistemic technologies, then their construction, diffusion, and stabilisation at broader institutional levels require further investigation. How do micro-level participation practices relate to the broader policy frameworks, funding structures, and professional networks that sustain them? How do the material and procedural scaffolds of participatory events interact with the normative and ideological commitments that underpin participation as a governance tool? These questions point to the need for an integrated perspective that links the micro and macro dimensions of participatory design, situating participation technologies within larger participation ecologies (Chilvers and Kearnes, 2020).

This expanded perspective offers a promising research agenda for STS scholars interested in participation. By treating participatory events as both structured and structuring, we can move beyond evaluations of participation as an ideal to be realised and instead investigate its situated practices, contestations, and effects in greater detail.

## Supplementary information

Supplementary Information

## Data Availability

The data collected for this study is qualitative, and due to the conditions set out in the study’s ethical approval and the consent obtained from research participants, it is not possible to share raw datasets outside of the research team. Given the media coverage of the assembly studied and the sample size used, data would likely be identifiable, even after anonymisation methods have been applied. The sharing of raw data, such as surveys, ethnographic notes, or interview transcripts, would risk compromising the legal and ethical commitments made to research participants and institutions that took part in the study. To support transparency, we provide a descriptive overview of the dataset used in this study, including: • Number of interviews: 11 interviews conducted in the period January 2023-May 2023. • Number of surveys: 25 surveys collected on 8th and 9th October 2022. • Ethnographic observation dates: Ethnographic fieldnotes were collected on 10 assembly event dates between May 2022 and March 2023. • Number of focus groups: 6 focus groups were conducted in the period December 2022-February 2023. Additionally, as supplementary material, we attach an extract of the coding template, developed for this study through the Template Analysis. Please note that the codebook is an extract of a wider codebook produced for a broader study, of which this paper focuses on one part, which may mean that parts of the sample book include occasional reference to examples not highlighted in the presented paper. The extract exemplifies the analytical structure of the study, outlining the development of key themes and including illustrative examples drawn from data collection, but without disclosing identifiable participant details. If additional clarification concerning the data collection and analytical procedures is needed by any researcher, they are welcome to contact the authors for further discussion.

## Footnotes

1 The assembly website is hosted by the Aarhus City Council rather than through an independent platform.

2 Foreword for the ACA recommendation report to the Aarhus City Council by its commissioning policymakers.
