# Supplementary material for: From structure to substance: public participation as a scaffolding technology in citizen assemblies
Source: Humanit Soc Sci Commun. 2026 Mar 19;13(1):630. doi: 10.1057/s41599-026-06965-y (PMC13167454; doi:10.1057/s41599-026-06965-y)
Supplement: Supplementary file 1 — Supplementary Information [file 41599_2026_6965_MOESM1_ESM.docx]

Appendix 1.

Example of coding schemes

| **Code** | **Meta-code family** | **Meaning** | **Reference** | **Example** |
| --- | --- | --- | --- | --- |
| 1.1 Agency | 1. Moral order | The ability to act' | [Joseph, J. (2018). agency. In A Concise Oxford Dictionary of Politics and International Relations. : Oxford University Press. From https://www.oxfordreference.com/view/10.1093/acref/9780199670840.001.0001/acref-9780199670840-e-1602.](https://www.oxfordreference.com/view/10.1093/acref/9780199670840.001.0001/acref-9780199670840-e-1602.) | For example, assembly members were presented with barriers by facilitators for discussing the full range of topics that they wished to include in their recommendations and which they believed to be within the political mandate of the assembly. They felt unable to act to include what they felt was important for the final recommendation report. |
| 1.2 Authority | 1. Moral order | Legitimate power, decision-making capacity, and the means to cause others to obey' | [authority. Oxford Reference. From https://www.oxfordreference.com/view/10.1093/oi/authority.20110803095435710.](https://www.oxfordreference.com/view/10.1093/oi/authority.20110803095435710.) | Facilitators using their authority as the organisers and designers of the process to guide, enable, or restrict the range and topics of citizen discussions during the assembly. |
| 1.3 Autonomy | 1. Moral order | Free will; self-governing, ability of a person or a group to choose a course of action' | autonomy. Oxford Reference. From https://www.oxfordreference.com/view/10.1093/oi/authority.20110803095436282. | Assembly members' experience of, at times, not having the free-will to include their subjective experiences in their deliberations due to the parameters of the OVA-structure and the expectation of objectivity. Equally, members' experiences of not having the free-will to include topics for consideration that they believed to be within the political mandate, but of which facilitators discouraged discussion. |
| 1.4 Boundary-making | 1. Moral order | The work done, through facilitation mechanisms and verbal guidance, by engagement practitioners to assert boundaries around citizens' participation in the engagement activities. | Drawing on Thomas Gieryn's work on "boundary-work". Gieryn, T. F. (1983). Boundary‑work and the demarcation of science from non‑science: Strains and interests in professional ideologies of scientists. American Sociological Review, 48(6), 781–795. | For instance, engagement practitioners stating that some topics are not included in the scope of engagement due to not being part of the political mandate, or being perceived by organisers as irrelevant to citizens' tasks in the engagement activity. |
| 2.1 Burden | 2. "Democratic innovation" | The work that is placed on citizens to enable climate engagement and action, specifically in contexts where this work is understood by citizens as work that is demanding of them. | Author | Some assembly members expressed frustration that they were being asked to spend time and effort on matters for which they felt local government should be responsible. Some members expressed that the process demanded more time and energy from them than had been communicated to them by organisers, as they felt they were required to undertake substantial amounts of independent research outside of the assembly meeting times to feel they could make informed and responsible contributions to the process. |
| 3.1 Citizen role | 3. Scaffolding | The role that citizens take on, or are expected to take on, in the engagement process, including how this role affects expectations for their contributions. | Author | For example, assembly members experienced a lack of clarity concerning their intended role in the process, including whether they were intended to identify new climate questions/issues, prioritise social action against climate action (including trade-offs in space and budget priorities for these), produce innovative suggestions or solutions for climate action, or sense-check existing climate policy plans. Strongly linked to "purpose". |
| 4.1 Community-driven | 4. Social ecology | Action, activities, or agendas led by the community | Author | For instance, where the community is enabled by organisers to have decision-making powers in identifying the aims, agenda, methods, and/or outputs of the engagement process. |
| 2.2 Consensus-making | 2. "Democratic innovation" | The process of achieving group consensus on a given question | Author | The climate assembly specifically aims to produce consensus on climate policy recommendations by the end of the assembly process, using a combination of deliberation and anonymous voting tools to achieve consensus. |
| 2.3 Deliberation | 2. "Democratic innovation" | Following the work of Dryzek and Mansbridge, deliberation is understood here to mean 'mutual communication that involves weighing and reflecting on preferences, values, and interests regarding matters of common concern'. | Bächtiger, A., Dryzek, J. S., Mansbridge, J., & Warren, M. E. (2018). Deliberative democracy: An introduction. In A. Bächtiger, J. S. Dryzek, J. Mansbridge, & M. E. Warren (Eds.), The Oxford handbook of deliberative democracy. Oxford University Press. https://doi.org/10.1093/oxfordhb/9780198747369.013.50 | The guidelines that assembly members were provided for their deliberation, which included the OVA-schedule and in-person facilitation, structured the ways in which citizens were enabled, supported, or prevented from expressing and weighing their perspectives, values, lived experiences, and knowledges regarding the assembly agenda topics. |
| 2. "Democratic innovation" | 2. "Democratic innovation" | The term democratic innovation covers all procedures aimed at facilitating and increasing citizens’ access and political participation, which are realized both through institutions specifically designed to increase public participation and through bottom-up experiences capable of providing connections to institutional practices in policy-making and political decision-making processes' | [Sorice, M. (2022). Democratic innovation. In P. Harris, A. Bitonti, C. S. Fleisher, & A. S. Binderkrantz (Eds.), The Palgrave encyclopedia of interest groups, lobbying and public affairs. Palgrave Macmillan. https://doi.org/10.1007/978-3-030-44556-0_66](https://doi.org/10.1007/978-3-030-44556-0_66) | Climate assemblies are widely regarded as a form of democratic innovation, aiming to facilitate greater citizen access to decision-making processes and increase the diversity of voices and lived experiences heard in the political process. These aspects of representation and access are reflected, for instance, through assemblies' sampling/selection methods for participants and in their aims to deliver citizen outputs directly to local government and policymakers. |
| 2.4 Democratic legitimacy | 2. "Democratic innovation" | Democratic legitimacy is understood in this context as the engagement process and its outcomes being regarded by policymakers and the public as being unbiased, reflecting the majority will of the assembly, and without undue influence by external forces or agendas, such as experts or policymakers. | Author | Ideas or concerns about engagement participations being unduly influenced in their recommendations by, for instance, experts who presented during the process, practitioners who sought to guide their work in particular directions within the scope of their mandate, or participants grounding their work in subjective political opinion rather than objective evidence as mandated by the OVA-structure. |
| 5.1 Disagreement | 5. Conflict | A situation in which people have different opinions, or an inability to agree' | Cambridge Dictionary. (n.d.). Disagreement. In Cambridge Dictionary online. From https://dictionary.cambridge.org/dictionary/english/disagreement | Instances where participants had different opinions about the nature of a problem or how to address it, seeing different positives and negatives associated with these, but not necessarily differences that were perceived as being incompatible, such as in the cases of different priorities |
| 5. Conflict | 5. Conflict | A situation in which beliefs, needs, facts, etc. are very different and cannot easily exist together or both be true' | [Cambridge Dictionary. (n.d.). Conflict. In Cambridge Dictionary online. From https://dictionary.cambridge.org/dictionary/english/conflict](https://dictionary.cambridge.org/dictionary/english/conflict) | For instance, conflicts arose between participants where their values, world views, or lived experiences did not align and were perceived, either initially or continuously, as being incompatible or unsuited for achieving consensus |
| 1. Moral order | 1. Moral order | "Moral order" draws on the work of Erving Goffmann and should be understood as the implicit or explicit rules of conduct communicated to participants, directly or indirectly, to indicate what is perceived to be morally acceptable behaviour within the metaphorical engagement space. | Goffman, E. (1967). Interaction ritual: Essays on face‑to‑face behavior. Anchor Books | An example of moral order from the climate assembly includes the use of the OVA-structure, which sets up implicit and explicit rules for how participants should structure their contributions, and, indirectly, their thinking, to conform to academic normative frameworks based on ideas of objectivity and evidence. Importantly, the communication and reinforcement of the OVA-structure leads to observations of engagement participants policying their own contributions and those of other participants when these are not perceived to align clearly with norms of, for instance, objectivity. |
| 3.2 OVA | 3. Scaffolding | The core working method that citizens were asked to structure their work around during the assembly, being short for "Observation, Assessment, Recommendation" ("Observation, Vurdering, Anbefaling" in Danish). The method was a tool that instructed citizens to work through each topic and question by following in order the three steps: i) Identification of the problem: '“Observation” is neutral—the objective problem'. ii) Delivering an assessment of the issue: '“Assessment” is how you understand the situation and find it important'. iii) Identifying a solution to the problem: '“Recommendation” is solution-oriented' (ACA Practitioner 1, o.n., 28.01.23). |  | The ACA practitioners stated that the OVA structure was intended to function as a ‘self-moderating tool’, encouraging participants to point out to one another when they would veer off from the deliberation level at hand—that is, observation, assessment, or recommendation—making the ‘conversation more democratic’ (ACA Practitioner 1, 2023). |
| 3.3 Outcomes | 3. Scaffolding | Understood to cover the planned outputs of the process, such as the climate policy recommendations, and the discussions about the exact nature of these. Also covers outcomes other than outputs, such as the engagement participants' experiences of participation in local governance and their perceptions of trust in policymakers and other involved engagement institutions. | Author | Understood to cover the planned outputs of the process, such as the climate policy recommendations, and the discussions about the exact nature of these. Also covers outcomes other than outputs, such as the engagement participants' experiences of participation in local governance and their perceptions of trust in policymakers and other involved engagement institutions. |
| 2.5 Performativity | 2. "Democratic innovation" | The perceived performative aspects of the engagement process and/or of the involvement of specific actors in the process. | Author | Some assembly members questioned the intentions of policymakers and other institutions involved in the process (such as the practitioners), expressing perceptions that some actors held alternative agendas for the involvement that were not aligned with the aims of the process. |
| 2.6 Policymakers | 2. "Democratic innovation" | The local policymakers from Aarhus City Council involved in either the commissioning, delivery, or processing of the the climate assembly. | Author | Representatives from the full range of local parties involved in the City Council were present at moments during the assembly and were also responsible for setting the mandate of the assembly. |
| 3.4 Practitioners | 3. Scaffolding | The practitioners involved in the assembly process included two sub-groups: 1) the facilitators, who were responsible for facilitating discussions in assembly sub-groups, but who did not influence the design of the process, including methods such as the OVA-structure. 2) The designers of the process who determined the structure, methods, aims, facilitation guidelines, and more of the process, and who led the plenary and voting sessions. Some members of this second group, at times, also led facilitation within sub-groups, making the overlap between the two groups significant, especially as perceived by the assembly participants. | Author | The practitioners involved in the assembly process included two sub-groups: 1) the facilitators, who were responsible for facilitating discussions in assembly sub-groups, but who did not influence the design of the process, including methods such as the OVA-structure. 2) The designers of the process who determined the structure, methods, aims, facilitation guidelines, and more of the process, and who led the plenary and voting sessions. Some members of this second group, at times, also led facilitation within sub-groups, making the overlap between the two groups significant, especially as perceived by the assembly participants. |
| 3.5 Process design | 3. Scaffolding | The process design included the selection of methods and exercises, the design of the relationships with experts, the system and composition of the sub-groups, the facilitation style and parameters, and the voting and consensus mechanisms embedded within the engagement process. | Author | As an example, the process design included specific choices of the OVA-structure, collective intelligence and creative exercises, a one-way "consulting" relationship with visiting experts during particular points of the process, and the choice to have assembly members vote anonymously and without group discussions on their final recommendations. |
| 3.6 Purpose | 3. Scaffolding | The purpose of the citizens' participation in the policy process and the intended nature of the policy recommendations with which they were tasked to produce. | Author | For instance, whether the policy recommendations are intended to be recommendations for priority areas for climate action, for acceptable trade-offs between, e.g. social and climate action and funding, or recommendations for innovative solutions to climate issues. |
| 3.7 Responsiveness | 3. Scaffolding | The practitioners' and other organsiers' (including policymakers') responsiveness to participant feedback during the engagement process. | Author | For example, the degree to which the practitioners responded to participants' ongoing confusion or criticism of the OVA-structure and their wishes for further discussions about the scope of their discussions. |
| 4. Social ecology | 4. Social ecology | The socio-cultural specific context of the engagement, including the historical relationships, network of social systems, cultural norms and values, and institutions that co-shape the space of the local community and how community members interact with the engagement process. | Author | A prominent example from the climate assembly of social ecology is the frequent reference from practitioners and participants to Grundtvigian norms and values around democracy. These ideas are invoked repeatedly by participants, such as through reference to civic duty and the right to question experts, but are subverted by practitioners (during data collection) in a stated effort to align the engagement scaffolding more closely with international standards than specific national socio-cultural norms. |
| 3. Scaffolding | 3. Scaffolding | Understood as the internal and external determination of the engagement process’ theme, aims, intended outcomes, scope, and definition of roles. |  | See, for instance, the details given elsewhere in the codebook under "purpose", "process design" and "citizen role". |
| 3.8 Tailoring | 3. Scaffolding | Tailoring the engagement process for the concrete assembly rather than using a "blueprint", standardised approach | Author | Tailoring includes aligning the process design with specificities of, for instance, the theme of the engagement (for example, climate action), the specific participants in the process, and/or the culture, norms, and social circumstances of the local community within which the engagement process takes place |
| 3.9 Transparency | 3. Scaffolding | Transparency in relation to the construction and aims of the engagement process | Author | Such as who influences the design of the process, the scope of citizens' decision-making powers, and mechanisms and commitments in place to process outcomes of the engagement process |
| 3.10 Trust | 3. Scaffolding | The feelings of trust between engagement participants and/or between participants and practitioners and/or between participants and engagement institutions, such as local policymakers | Author | For example, some assembly members stated that they felt less trust in policymakers because they experienced the assembly process as not having clear aims for their involvement, leading, for some, to the impression that the process was performative and their recommendations would not be treated meaningfully |
| 1.5 Uncertainty | 1. Moral order | Uncertainty is understood as the experience that something is not known or certain in the context of deliberation and decision-making | Author | For instance, the uncertainty about the purpose and role of citizens' contributions, leading to confusion about the expectations for their existing knowledge about the subject matter, the degree to which they could include subjective, lived experience, and whether they were intended to produce, e.g. climate action solutions or prioritise social versus climate action |
